# Perceptions Toward Established and Novel Dietary Therapies for Crohn’s Disease Management Among Adult Patients: Results From a Questionnaire Survey

**DOI:** 10.1093/crocol/otae008

**Published:** 2024-02-06

**Authors:** Aleksandra Jatkowska, Bernadette White, Paige Jaskolski, Ben Nichols, Emily Brownson, Jennifer Clowe, John Paul Seenan, Konstantinos Gerasimidis, Jonathan MacDonald

**Affiliations:** Human Nutrition, School of Medicine, Dentistry and Nursing, University of Glasgow, Glasgow, UK; Human Nutrition, School of Medicine, Dentistry and Nursing, University of Glasgow, Glasgow, UK; Human Nutrition, School of Medicine, Dentistry and Nursing, University of Glasgow, Glasgow, UK; Human Nutrition, School of Medicine, Dentistry and Nursing, University of Glasgow, Glasgow, UK; Human Nutrition, School of Medicine, Dentistry and Nursing, University of Glasgow, Glasgow, UK; Department of Gastroenterology, Queen Elizabeth University Hospital, Glasgow, UK; Human Nutrition, School of Medicine, Dentistry and Nursing, University of Glasgow, Glasgow, UK; Department of Gastroenterology, Queen Elizabeth University Hospital, Glasgow, UK; Human Nutrition, School of Medicine, Dentistry and Nursing, University of Glasgow, Glasgow, UK; Department of Gastroenterology, Queen Elizabeth University Hospital, Glasgow, UK; Human Nutrition, School of Medicine, Dentistry and Nursing, University of Glasgow, Glasgow, UK; Human Nutrition, School of Medicine, Dentistry and Nursing, University of Glasgow, Glasgow, UK; Department of Gastroenterology, Queen Elizabeth University Hospital, Glasgow, UK

**Keywords:** Crohn’s disease, dietary therapies, enteral nutrition, exclusion diets, patient-centered care

## Abstract

**Background:**

Exclusive enteral nutrition (EEN) and partial enteral nutrition (PEN) remain the only established dietary therapies in Crohn’s disease (CD) management. We conducted a questionnaire survey to evaluate the perceptions of adults with CD toward established and emerging food-based dietary therapies.

**Methods:**

A 26-question anonymous survey was mailed to 300 adults receiving biologic treatment. Two researchers independently conducted a thematic analysis of open-ended responses. Machine learning with the Random Forest–Recursive Feature Elimination algorithm identified predictors of willingness to try dietary therapies.

**Results:**

One hundred and sixty patients (53% female) completed and returned the survey. Forty-two percent were following some form of exclusion diet, with low-spice and low-fiber diets being the most popular. Although only a quarter of patients believed that EEN/PEN could help with their CD, more than half believed that diet could help, with another 13% already using diet for CD management. While half of the patients were willing to try EEN, the majority were willing to try PEN instead (51% vs. 79%; *P* < .001). Forty-two percent of patients preferred food-based dietary plans prepared at home over EEN/PEN options. The most important predictors for willingness to try dietary therapies were age (25–65 years), recent symptoms, previous exposure to EEN/PEN, and current exclusion diet use. The top concerns about PEN were taste/palatability, satiety/hunger, and taste fatigue.

**Conclusions:**

Most adults preferred to follow a food-based dietary therapy over EEN/PEN. The majority would try PEN though which allows for more flexibility to incorporate in habitual diet and may be easier to comply with than the EEN.

## Graphical Abstract

**Figure:**
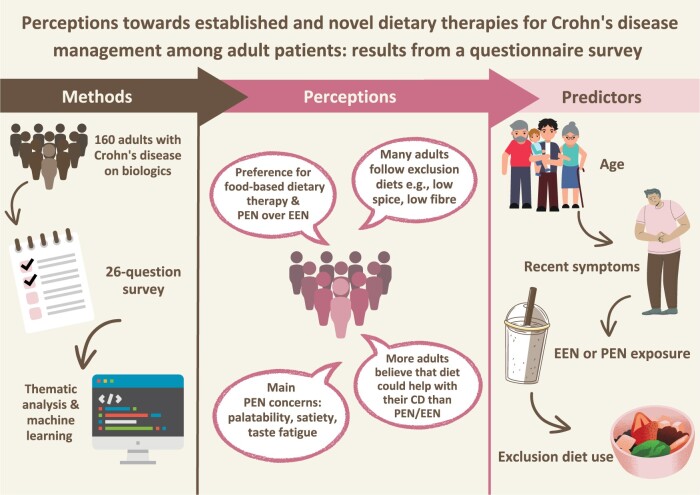

## Introduction

While genetic factors contribute to the etiology of Crohn’s disease (CD),^1^ nutritional epidemiology suggests that the rising global prevalence of the condition cannot solely be attributed to genetic factors.^2^ Rather, it is believed that exposure to environmental factors, such as western dietary patterns and gut microbiome dysbiosis, in individuals with genetic susceptibility, are the main contributors to the rising prevalence of CD.^3–6^ While there is currently no cure for CD, various treatments are used to induce and maintain disease remission. These treatments include corticosteroids, biologics, and dietary therapies such as exclusive enteral nutrition (EEN) and partial enteral nutrition (PEN). Corticosteroids suppress the immune system indiscriminately, whereas biologics, such as antitumor necrosis factor (anti-TNF) drugs, target specific inflammatory pathways, making them a more desirable treatment option for CD management. However, the primary response rates to biologics are suboptimal, at approximately 40%–50%, with the potential for a subsequent 10%–20% secondary loss of response rate for each year of treatment.^7^ Patients who have a partial or suboptimal treatment response may require dose escalation or combination therapy with immunomodulators to establish better disease control.^8^ However, immunomodulators have the potential to cause significant adverse effects.^9^

Established dietary therapies, particularly EEN and PEN, have gained recognition in the management of CD due to their high effectiveness and favorable safety profile. Treatment of active CD with EEN has at least comparable efficacy to oral corticosteroids, a superior safety profile,^10,11^ and additionally promotes mucosal healing and improves nutritional status.^12,13^ Despite its mainstream use as first-line treatment in the pediatric population, in Europe and elsewhere, use of EEN is less frequent among adults primarily due to challenges related to poor compliance, taste fatigue, palatability issues, and limited dietetic resources in adult gastroenterology departments.^14^ In contrast, PEN, which replaces only part of habitual diet with formula, may be easier to adhere to and has a good record of efficacy as a standalone maintenance treatment in pediatric patients; particularly when it replaces a high part of a person’s habitual diet. Studies, primarily from Japan, have suggested that PEN may also be effective in adult patients, particularly when used in combination with biological treatments^15^ and at proportions above 35%–50% of energy requirements.^16^

Despite the high effectiveness of EEN in managing CD, there are also concerns about its negative impact on the social interactions and quality of life of patients and their families.^14^ Nonetheless, a survey of children who received EEN treatment revealed that 59% of children were willing to undergo another course to manage future disease relapses.^17^ Most children (66%) and their parents (72%), though, preferred a food-based dietary therapy as an alternative to EEN, if available as an option. In recent years, there has been a growing interest in exploring food-based dietary therapies for managing CD. Two such emerging therapies are the Crohn’s disease treatment-with-eating (CD-TREAT) diet and the Crohn’s Disease Exclusion Diet (CDED) coupled with 50% PEN. The CD-TREAT diet is a food-based regimen that mimics EEN by matching its nutrient content and excluding the same nutrients (lactose, gluten, and alcohol),^18^ whereas the CDED is based on epidemiological data and animal experiments that recommends, mandates, and excludes certain food products.^19,20^ Despite the recent advancements in this area, EEN and PEN, replacing at least 50% of habitual diet with formula, remain the only evidence-based dietary therapies supported by guidelines from major inflammatory bowel disease (IBD) associations.^10^ While current evidence suggests that novel dietary therapies hold a promise in the management of adult CD, there is limited data on patient perceptions toward such therapies, especially PEN replacing 50% of the habitual diet—an approach which might be more acceptable in this patient population.

The main objective of the present study was to investigate the perceptions and beliefs of adults with CD receiving biological therapy toward established dietary therapies, including EEN, PEN replacing 50% of habitual diet, and novel emerging food-based dietary therapies. This study also aimed to identify determinants that influence their willingness to adopt each of the dietary therapies. Last, we assessed patient concerns regarding the use of PEN and evaluated patient preferences concerning the adoption of different dietary therapies.

## Methods

### Survey Development

A 26-question anonymous survey was developed by adult gastroenterologists from the National Health Service in Greater Glasgow & Clyde and nutrition researchers from the University of Glasgow with expertise in IBD research (copy available at DOI: 10.5525/gla.researchdata.1481). The content of the survey was put together and checked by other members of the medical and research teams to ensure that it is reflective of patients’ concerns and practice, and readability was assessed by layperson review. The survey collected information about demographics, eating habits (current use of exclusion diets, eating the same or different meals as the rest of household, food preparation), self-reported disease characteristics, and perceptions and beliefs toward dietary therapies including EEN, PEN replacing 50% of energy requirements, and food-based dietary therapies for CD management. The survey consisted of a range of single-choice, multiple-choice, and open-ended questions. Two open-ended questions were asked about challenges, perceptions, and concerns about the use of PEN.

### Recruitment

Adult patients (>16 years) with a diagnosis of CD treated with biologics by the gastroenterology teams within the National Health Service in Greater Glasgow & Clyde were identified by administrative staff of the clinical team. Anonymized surveys with prepaid return envelopes were mailed to the first 300 identified patients in November 2021. A reminder was sent in February 2022, including a note asking patients who had already completed the survey, to discard the reminder survey, and not complete and return it again.

### Thematic Analysis

Answers to the open-ended questions were analyzed independently by 2 researchers using inductive thematic analysis with answers grouped into common categories. The results were compared, and any discrepancies were resolved through a consensus discussion between the 2 researchers (A.J. and P.J.). In case of a disagreement, a third independent researcher was consulted to reach a final consensus (B.W.). Themes raised by less than 5% of the responders were excluded from the thematic analysis.

### Data Analysis

Data analyses were performed in Minitab Version 19 (Minitab Ltd, Coventry, UK) and R version 4.1.2. Categorical data were presented with counts and frequencies (%). The associations between categorical variables were tested using chi-square test and post hoc Fisher’s exact test, when appropriate. Responses such as incorrect answers, no answers, and “Prefer not to say” were excluded from all questions. Additionally, responses with low frequency (<5) were removed, and some responses were grouped together when deemed appropriate to gain statistical power in downstream statistical analyses (Supplementary Table 1). The most relevant predictors, for willingness to try different dietary therapies and beliefs about their effectiveness, were selected using two approaches: (1) Comparisons between categories using chi-square test with post hoc Fisher’s exact test, and (2) machine learning, specifically with the Random Forest–Recursive Feature Elimination (RF-RFE) algorithm, first by removing features that did not contribute to the overall performance of the Random Forest (RF) using the “FeatureTerminatoR” R package^21^ and then generating RF models using the “randomForest” R package on the reduced set of features. The number of decision trees was set to 50 000 and the models were trained and validated using the bootstrap aggregation procedure implemented within the RF algorithm, whereby performance is measured using the “out-of-bag” (OOB) samples, that is, those records that were not included in each decision tree during each iteration of bootstrap aggregation. The Gini impurity index measures the impurity or disorder of the data, and it helps determine the importance of selected predictors by measuring how much they individually contribute to reducing the disorder of categories in the data. We then evaluated the performance and generalizability of the machine learning models by calculating the OOB error rate. A variety of demographic characteristics, disease features, and eating habits were considered as potential predictors. Model significance was assessed using a permutation test implemented in the “rf.significance” function in the “rfUtilities” R package.^22^Supplementary Table 2 lists the predictors that were examined. Statistical significance was set at *P* < .05.

### Ethical Permissions

Caldicott guardian approval for the distribution of the final version of the questionnaire was obtained from the National Health Service in Greater Glasgow and Clyde.

## Results

A total of 300 adult patients with CD receiving biologic therapy were identified and sent the survey, along with a reminder. Out of these, 160 patients completed and returned the survey, resulting in a response rate of 53%. Among the 160 patients, 121 (76%) completed all 24 questions (excluding the 2 optional open-ended questions), demonstrating a high level of answer completeness. Moreover, all patients responded to at least 79% of the 24 questions, indicating a high level of engagement. The study cohort consisted primarily of Caucasian patients (96%, 154/160) aged between 25 and 39 years (29%, 46/160). The majority of patients had been diagnosed with CD over a decade ago (57%, 91/159), reported experiencing at least 1 CD-related symptom in the last 7 days (74%, 116/156), and rated their current disease activity as mild to moderate (61%, 90/148). The demographic, disease, and treatment characteristics of patients who returned the survey are presented in Supplementary Table 3.

### Eating Habits

**Figure 1.**
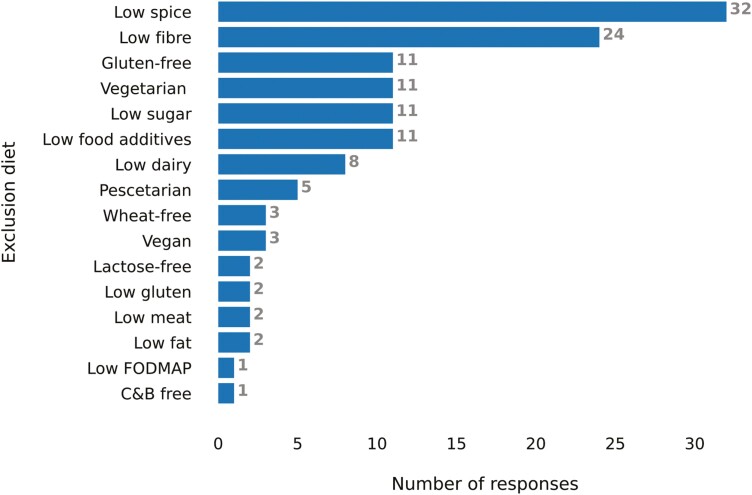
Exclusion diet use among 67 adult patients with Crohn’s disease currently following exclusion diets. C&B, Cinnamon and Benzoates; FODMAP, Fermentable Oligosaccharides, Disaccharides, Monosaccharides and Polyols.

The majority of patients reported preparing their own meals (64%), while only a small number of patients primarily relied on takeaway/fast food (1%), ready microwave meals (1%), or restaurant food/dining out (1%). Most patients (63%) reported eating the same meals as the rest of their household (63%), and 1 in 5 patients (18%) had previously used a liquid meal replacement for reasons other than CD management (Supplementary Table 4). Interestingly, 42% (67/158) of patients reported currently following an exclusion diet, with low-spice and low-fiber diets being the most common types (Figure 1). Patients with previous or current PEN or EEN use (38% of patients) indicating prior dietetic input were more likely to follow a low-fiber diet (*P* = .040), be willing to try EEN (*P* = .019), and believe that PEN or EEN could be effective to manage their disease (*P* = .047).

### Patient Perceptions Toward Dietary Therapies for CD Management

**Figure 2.**
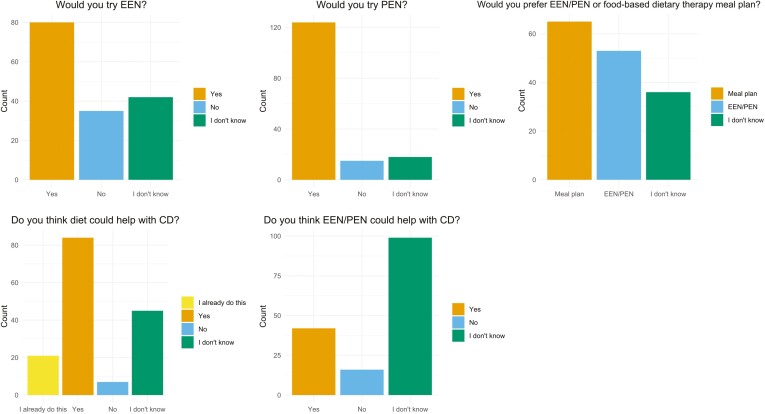
Patient perceptions and beliefs towards dietary therapies for Crohn’s disease management. CD, Crohn’s disease; EEN, exclusive enteral nutrition; PEN, partial enteral nutrition.

Half of the patients (51%) reported willingness to try EEN, while nearly 4 out of 5 patients (79%) were willing to try PEN (EEN vs. PEN: *P* < .001). However, when given a choice between EEN/PEN and a food-based dietary therapy, 42% of patients preferred to follow a food-based dietary therapy. More than half of the patients (54%) believed that diet could help with their CD, with an additional 13% of patients already using diet for CD management. Conversely, only a quarter of the patients (27%) believed that EEN and PEN could be effective for CD management, and more than two-thirds of patients were uncertain about the effectiveness of these treatments (“I don’t know,” 63%) (Figure 2).

### Predictors of Willingness to Try Dietary Therapies and Patient Beliefs About Their Effectiveness

**Figure 3.**
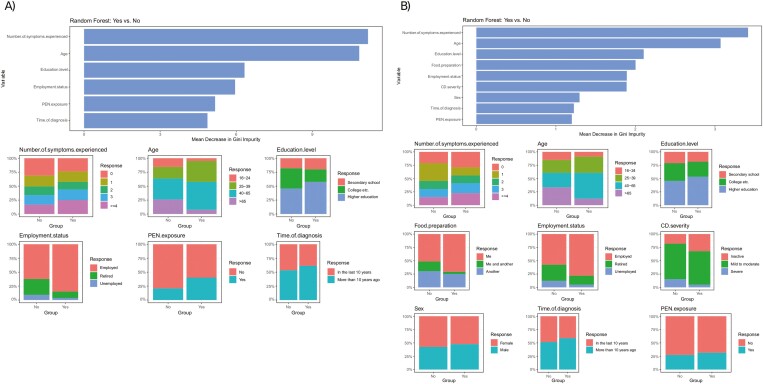
Results of random forest analysis with mean decrease in Gini impurity score for (A) willingness to try exclusive enteral nutrition (area under the curve: 0.736; out-of-bag error: 0.32; *P*-value: <.001), and (B) willingness to try partial enteral nutrition (area under the curve: 0.512; out-of-bag error: 0.33; *P*-value = .001). CD, Crohn’s disease; PEN, partial enteral nutrition.

Willingness to try EEN was influenced by age, employment status, and previous/current use of PEN. Specifically, patients aged 16–24 and over 65 years were less willing to try EEN compared to those aged 25–65 (16–24 vs. 25–39: *P* = .0082; 16–24 vs. 40–65: *P* = .0254; >65 vs. 25–39: *P* = .0012; >65 vs. 40–65: *P* = .0028). Retired patients were also less willing than employed patients (*P* = .0067). Conversely, patients with previous/current PEN use were more likely to try EEN (*P* = .0126). Machine learning with the RF-RFE algorithm confirmed age and the number of symptoms experienced in the last 7 days as the most important predictors for EEN willingness (Figure 3A).

Like with EEN, age and household food preparation role were significant factors for willingness to try PEN. Patients over 65 were less likely to try PEN compared to those aged 25–65 (>65 vs. 25–39: *P* = .0279; >65 vs. 40–65: *P* = .0038). Patients sharing food preparation with a household member were also less willing than those preparing their own meals (me and another household member vs. me: *P* = .0087). RF-RFE analysis algorithm highlighted age (25–65 years) and the number of symptoms experienced in the last 7 days as the most important predictors for PEN willingness (Figure 3B).

**Figure 4.**
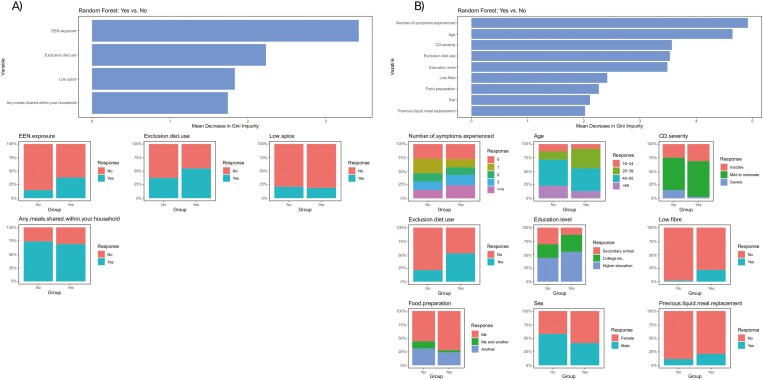
Results of random forest analysis with mean decrease in Gini impurity score for (A) belief that enteral nutrition could help with Crohn’s disease management (area under the curve: 0.626; out-of-bag error: 0.38; *P*-value = .001), and (B) belief that diet could help with CD management (area under the curve: 0.745; out-of-bag error: 0.25; *P*-value: <.001). CD, Crohn’s disease.

Belief in the effectiveness of EEN and PEN for CD management was higher among patients with previous/current EEN use (*P* = .0035), a finding which was also confirmed with the RF-RFE algorithm (Figure 4A). Age and current exclusion diet use were also significant predictors for the belief that diet could help with CD management. Patients aged 25–39 years were more likely to hold this belief compared to those aged 16–24 (*P* = .0463), 40–65 (*P* = .0357), and over 65 (*P* = .0135). Similarly, patients following an exclusion diet (*P* = .0004) and specifically a low-fiber diet (*P* = .0025) were more likely to hold this belief. In contrast, patients who rated their disease activity as severe (severe vs. inactive: *P* = .0122; severe vs. mild to moderate: *P* = .0153) and those sharing food preparation with another household member (me and another household member vs. me: *P* = .0339) were less likely to believe in the effectiveness of diet for CD management. The RF-RFE algorithm identified age and the number of symptoms experienced in the last 7 days as the most significant predictors for this belief (Figure 4B).

### Concerns Regarding the Use of PEN: Thematic Analysis

**Figure 5.**
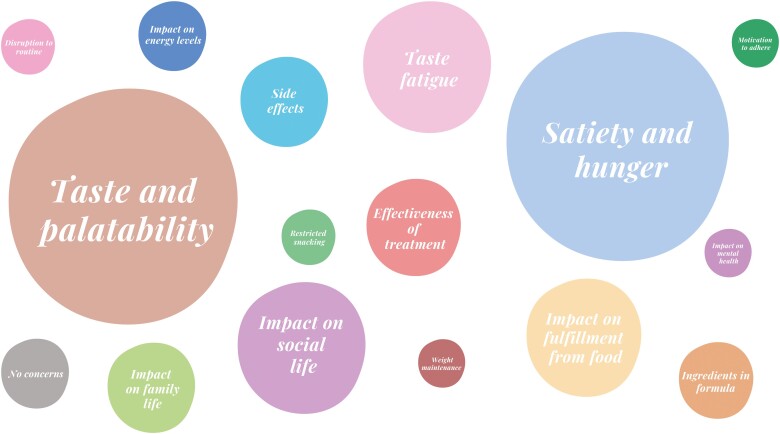
Top concerns (≥5% response rate) reported by adult patients with Crohn’s disease regarding partial enteral nutrition use (*n* = 136). The size of each bubble and the font size within each bubble represents the number of responses matching each category.

Of the 160 patients who completed and returned the survey, the vast majority (92%, 147/160) responded to at least 1 open-ended question about potential challenges or concerns they may have if asked to follow PEN for CD management. Of those, 11 patients (7%) provided incorrect/implausible responses and were excluded from the analysis, leaving 136 valid responses. Thematic analysis of these responses revealed 24 distinct themes of concerns, with the most frequently mentioned being taste/palatability (25%), satiety/hunger (22%), taste fatigue (14%), and the impact on social life (14%). Only a minority of responders (7%) reported having no challenges or concerns regarding the use of PEN for CD management (Figure 5, Supplementary Table 5).

### Patient Preferences Concerning the Implementation of Dietary Therapies for CD Management

A meal plan that allows flexibility to patients to customize meal replacements with PEN was preferred by the majority of patients. However, when presented with the option to choose a single meal they would not like to change with PEN, most patients opted to keep dinner (Table 1). Patients over 65 and those with higher education were more inclined to favor a meal plan that maintained consistency in replacing the same meals daily (Supplementary Figure 1).

**Table 1.** Patient preferences concerning the administration of dietary therapies for disease management.

Questions	Answers, % (*n*)		
	Breakfast	Lunch	Dinner
Which one meal would you like to keep as solid food during PEN?	14% (21/149)	12% (18/149)	74% (110/149)
Which one meal would you like to keep as solid food during PEN?^a^	12% (14/121)	12% (14/121)	77% (93/121)

	Morning snack	Afternoon snack	Evening snack
Which one snack would you like to keep as solid food during PEN?	28% (39/136)	37% (50/136)	35% (47/136)
Which one snack would you like to keep as solid food during PEN?^a^	30% (34/114)	35% (40/114)	35% (40/114)

	Strict meal plan	Flexible meal plan	I don’t know
Would you prefer a strict or flexible meal plan during PEN?	29% (45/153)	60% (92/153)	10% (16/153)
Would you prefer a strict or flexible meal plan during PEN?^a^	30% (37/123)	66% (81/123)	4% (5/123)

	Premade/ready-to-drink milkshake	Milkshake powder	I don’t know
Would you prefer a premade/ready-to-drink milkshake or milkshake powder to prepare yourself during EEN or PEN?	62% (95/153)	21% (32/153)	17% (26/153)
Would you prefer a premade/ready-to-drink milkshake or milkshake powder to prepare yourself during EEN or PEN?^b^	64% (79/124)	23% (28/124)	14% (17/124)

	Meal plan to prepare at home	Meal plan with premade meals delivered	I don’t know
Would you prefer a food-based dietary therapy with meal plan to prepare at home or with premade meals delivered?	50% (76/151)	39% (59/151)	11% (16/151)

^a^Subgroup analysis including only patients who would try PEN (*n* = 124).

^b^Subgroup analysis including only patients who would try either EEN or PEN (*n* = 125).

Abbreviations: EEN, exclusive enteral nutrition; PEN, partial enteral nutrition.

When it comes to formula preferences during PEN or EEN treatment, most patients preferred premade/ready-to-drink formulas (Table 1). Patients who experienced at least 1 symptom in the last 7 days (*P* = .0476) or had undergone intestinal resection (*P* = .0052) were more likely to prefer a premade/ready-to-drink formula. This preference was also observed in patients who rated their disease activity as mild to moderate compared to those with inactive or severe disease activity (mild to moderate vs. inactive: *P* = .0407; mild to moderate vs. severe: *P* = .0017). The RF-RFE algorithm confirmed that the number of symptoms experienced in the last 7 days and previous intestinal resection were the most significant predictors (Supplementary Figure 2).

Regarding food-based dietary therapies, most patients preferred preparing their meals at home (50%) rather than having premade meals delivered (Table 1). Patients with higher education (higher education vs. college: *P* = .0010; higher education vs. secondary school or lower: *P* = .0445) and those who rated their disease activity as inactive (inactive vs. mild to moderate: *P* = .0114) were more inclined to choose to prepare meals at home. In contrast, patients who experienced at least 1 symptom in the last 7 days (symptoms vs. no symptoms: *P* = .0003; 0 symptoms vs. 1 symptom: *P* = .0080; 0 symptoms vs. 2 symptoms: *P* = .0070; 0 symptoms vs. 3 symptoms: *P* = .0008; 0 symptoms vs. ≥4 symptoms: *P* = .0064), those who previously used liquid meal replacement for a reason other than disease management (*P* = .0004), and those who tried EEN (*P* = .0062) preferred a food-based dietary therapy with premade meals delivered. The RF-RFE algorithm identified the number of symptoms experienced in the last 7 days and education level as the most important predictors for the preference for either preparing their own meals or opting for premade meal delivery during food-based dietary therapies (Supplementary Figure 3).

## Discussion

This study explored the perceptions of adult patients regarding established and novel dietary therapies for the management of CD, as well as their general perceptions regarding the role of diet in CD. Despite only 51% of respondents expressing willingness to try EEN, a much greater proportion were open to trying PEN, which may be more practical for adults, assuming not much inferior effectiveness. Positive attitudes toward liquid nutritional therapy with EEN or PEN were associated with age, showing an optimal range where individuals were neither too young nor too old, as well as with more recent symptoms. These findings are of significance as they can assist healthcare professionals in stratifying treatment decisions and identifying patient groups that require increased awareness regarding the role of diet in CD management. Treatment with EEN or PEN involves significant restrictions on habitual diet, and the results of the current study suggest that these dietary regimes become treatment options for patients with active disease who might be more likely to accept dietary restrictions. Supporting this observation is the volume of literature and clinical experience where EEN is commonly used as a first-line therapy in children with CD, while in adult patients, it is used as a rescue therapy in treatment-refractory patients and prior to major gastrointestinal surgery.

Interestingly, the majority of patients in this study demonstrated a preference for food-based dietary therapies as opposed to PEN or EEN. This finding aligns with a previous survey conducted on children who had previously undergone EEN treatment.^17^ Consistent with the results of that study in children, adult patients who had previous experience with EEN or held positive views regarding the role of diet in CD management exhibited higher levels of acceptance towards treatment with EEN or PEN; hence reinforcing the need for education on the importance of diet in IBD management.

Most patients in the current study held the belief that diet is an effective approach to managing their CD. This finding is consistent with a previous study involving 294 adults with IBD, which reported that over 60% of patients achieved symptom management through dietary modifications, although it remains unclear whether this included EEN or PEN treatments.^23^ In contrast, a significant proportion of patients in our study were uncertain regarding the effectiveness of EEN and PEN, despite more evidence supporting their clinical efficacy than with the use of food-based dietary therapies. This could be attributed to the limited exposure to these treatments in the adult patient population, as they are not widely supported as treatment options in adult CD within the IBD guidelines,^24^ and adults have access to less dietetic support, nutritional education, and resources compared to children with CD.^25^ Patients with exposure to EEN were more inclined to believe in the potential of EEN and PEN to aid in CD management and patients aged 25–65 who followed an exclusion diet, specifically a low-fiber diet, were more likely to hold the belief that diet could help with their CD, hence reinforcing the needs for nutritional education of IBD patients.

Dietary exclusions are prevalent among patients with IBD, and the findings of the current study are in accordance with previous research.^26^ Prior findings indicated that patients with IBD consider spicy foods, high-fat foods, raw vegetables, fizzy soft drinks, high-fiber foods, dairy products, and coffee or tea as risk factors triggering disease relapses. In accordance with this previous evidence, the present study also observed that many patients were following exclusion diets, with low-spice and low-fiber diets being the most popular among adult patients with CD. Although details regarding prior dietetic input were not collected in the current study, exposure to PEN and EEN was used as a proxy of dietary education and patients with exposure to PEN and/or EEN were more likely to be following a low-fiber diet.

Furthermore, the current study gathered information on patient preferences regarding the adoption of various dietary therapies, including PEN, EEN, and food-based dietary therapies. These findings can assist healthcare professionals in tailoring treatments to better suit individual patient needs. The majority of patients in our study expressed a preference for premade/ready-to-drink formulas, particularly among those who had experienced symptoms within the past 7 days or had undergone intestinal resection. The convenience of ready-to-drink formulas is likely to be advantageous, particularly for patients who experience disease-related fatigue or have severe disease activity. Considering patient preferences in dietary therapy is crucial and the current study emphasized the importance of flexibility around PEN use, allowing for the customization of habitual diet meal replacements based on patient’s lifestyle. This flexibility can enhance treatment adherence and, by extension, treatment effectiveness. In contrast, regarding the use of food-based dietary therapies for CD treatment, the majority of patients preferred preparing their meals at home. This preference suggests that our responders exhibit characteristics linked to a positive attitude toward dietary therapy for CD. They appear to be health-conscious individuals who find satisfaction in preparing food and engaging in dining experiences. However, patients who had experienced more symptoms in the past 7 days were more likely to prefer to have meals delivered rather than prepare them at home. This preference also aligns with their preference toward the use of premade/ready-to-drink formula with PEN or EEN.

Last, this study has highlighted several concerns that patients have regarding the use of PEN, including issues with palatability and taste fatigue of the formula, a sense of not feeling satiated, and concerns about how it may affect their social life. As food plays a vital role in an individual’s social interactions and lifestyle, ensuring that patients receive adequate dietetic support throughout their treatment is crucial.

This work has certain limitations that need to be considered when interpreting the findings. First, the generalizability of the study may be limited as only patients on biologic treatment from a single health board were invited to participate. The majority of responders were of Caucasian background, and results may not be generalizable to other populations and countries. It is also noteworthy that over 40% of the responders had completed higher education, and a majority expressed a preference for preparing their own meals with only a minority relying on takeaway/microwave meals. These demographics may not be representative of the Scottish population of adults with CD, potentially introducing a sampling bias that should be taken into consideration when interpreting the findings. Since this survey was anonymous, we are also unable to compare the characteristics between responders and non-responders to this survey. Moreover, we did not collect data on concerns about EEN use to minimize participant burden, although previous research has investigated this.^14^

In conclusion, the current study found that adults with CD displayed a greater willingness to try PEN and food-based dietary therapies, compared to EEN. This highlights the clinical need for considering the use of PEN as a treatment option in this patient population and emphasizes the need for further research in the area of food-based dietary therapies, which are currently not widely recommended in the management of CD. We also identified several factors that can predict a patient’s willingness to try different dietary therapies, which can be used to identify patients who are more likely to accept this type of therapy and stratify treatment options accordingly. In contrast, patients who lack awareness or fail to recognize the significance of nutritional therapy as a viable treatment option in CD should be the focus of targeted nutrition education efforts.

## Supplementary Material

otae008_suppl_Supplementary_Tables_1-5_Figures_1-3

## Data Availability

Datasets will be shared upon request.

## Publication Statement

We confirm that this manuscript including related data, figures, and tables has not been previously published and is not currently under consideration elsewhere.
