# Supplementary material for: Perceptions Toward Established and Novel Dietary Therapies for Crohn’s Disease Management Among Adult Patients: Results From a Questionnaire Survey
Source: Crohns Colitis 360. 2024 Feb 6;6(1):otae008. doi: 10.1093/crocol/otae008 (PMC10924435; doi:10.1093/crocol/otae008)
Supplement: otae008_suppl_Supplementary_Tables_1-5_Figures_1-3 [file otae008_suppl_supplementary_tables_1-5_figures_1-3.docx]

| **Question** | **Original grouping** | **Data cleaning performed** | **Grouping post data clean** |
| --- | --- | --- | --- |
| Age | <18, 18-24, 25-39, 40-65, >65 | - “<18” (n=1) merged with “18-24” (n=15) -> “16-24” | 16-24, 25-39, 40-65, >65 |
| Living situation | Living alone, Living with a partner, Living with family members, Living in shared accommodation | - “Living alone” (n=34) merged with “Living in a shared accommodation” (n=7) -> “Living without family members”  - “Living with family members” (n=56) merged with “Living with a partner” (n=62) -> “Living with family members”  - No answer (n=1) removed | Living without family members, Living with family members |
| Education level | No schooling completed, Primary school, Secondary school, College/Sixth form or equivalent, Trade/technical/vocational training, Bachelor’s degree, Master’s degree, Doctorate degree, Prefer not to say | - “No schooling completed” (n=4) and “Primary school” (n=1) merged with “Secondary school” (n=26) -> “Secondary school or lower”  - “College/Sixth form” (n=29) merged with “Trade/technical/vocational training” (n=18) -> “College/Sixth form/Trade/technical/vocational training”  - “Master’s degree” (n=21) and “Doctorate degree” (n=8) merged with “Bachelor’s degree” (n=41) -> “Higher education”  - “Prefer not to say” (n=11) and no answer (n=1) removed | Secondary school or lower, College/Sixth form/Trade/technical/vocational training, Higher education |
| Employment status | In full-time employment, in part-time employment, Self-employed, In full-time education, In part-time education, Stay-at-home parent/homemaker, Retired, Unemployed, Prefer not to say | - “Part-time employment” (n=23) and “Self-employed” (n=13) merged with “Full-time employment” (n=62) -> “Employed”  - “Prefer not to say” (n=3) and “Stay-at-home parent” (n=3) removed | Employed, Unemployed, Retired |
| Educational status | In full-time education, In part-time education, Prefer not to say | - “Full-time education” (n=14) merged with “Part-time education” (n=3) -> “In education”  - “Prefer not to say” (n=3) removed | In education, Not in education |
| Ethnicity | White British, White Irish, White Other, Pakistani, Asian Other, White and Asian White and Black Caribbean | - “Irish White” (n=4) and Other White (n=9) merged with “British White” (n=141) -> “Caucasian”  - “Other Asian” (n=1), “Pakistani” (n=3), “White and Asian” (n=1) merged with “White and Black Caribbean” (n=1) -> “non-Caucasian” | Caucasian, Non-Caucasian |
| Do you follow any exclusion diet? | Yes, No | - No answer (n=2) removed | Yes, No |
| Have used liquid meal replacement outside CD? | Yes, No, | - No answer (n=3) removed | Yes, No |
| Food preparation | Me, My partner, Another household member, Me and another household member, I mostly eat ready microwave meals, I mostly eat takeaway/fast food, I mostly eat restaurant food/dine out, Prefer not to say | “Another household member” (n=14) merged with “My partner” (n=27) -> “Another household member”  - “I mostly eat ready microwave meals” (n=1), “I mostly eat takeaway/fast food” (n=2), “I mostly eat restaurant food/dine out”, “Prefer not to say” (n=1) and no answer (n=3) removed | Me, Another household member, Me and another household member |
| Do you eat the same meals? | Yes, No, Not applicable/I live alone | - “No” (n=28) merged with “Not applicable/I live alone” (n=28) -> “No”  - No answer (n=8) removed | Yes, No |
| Any meals/snacks shared? | Yes, No, Not applicable/I live alone | - “No” (n=13) merged with “ Not applicable/I live alone” (n=32) -> “No” | Yes, No |
| How many meals/snacks shared | 0, 1, 2, 3, 4, 5, 6, Not applicable/I live alone | - “0” (n=13) merged with “Not applicable/I live alone” (n=32) -> “0”  - “3” (n=38), “4” (n=2), and “5” (n=4) merged with “6” (n=3) -> “≥3” | “0”, “1”, “2”, “≥3” |
| Treatment history | EEN, PEN, Steroids, Immunosuppressants, Intestinal resection | - No answer (n=1) removed | EEN, PEN, Steroids, Immunosuppressants, Intestinal resection |
| Symptoms experienced in the last 7 days? | 0, 1, 2, 3, 4, 5, >5 | - “4” (n=21) and “5” (n=9) merged with “>5” (n=3) -> “≥4”  - No answer (n=4) removed | 1, 2, 3, ≥4 |
| CD severity, self-reported | Inactive, Mild to moderate, Severe, I don’t know, Prefer not to say | - “I don’t know” (n=10), “Prefer not to say” (n=1) and no answer (n=1) removed | Inactive, Mild to moderate, Severe |
| Time of diagnosis | Last year, In the last 5 years, In the last 10 years, More than 10 years ago | - “Last year” (n=5) and “In the last 5 years” (n=35) merged with “In the last 10 years” (n=28) -> “In the last 10 years”  - No answer (n=1) removed | In the last 10 years, More than 10 years ago |
| Which one meal would you like to keep as solid? | Breakfast, Lunch, Dinner | - No answer (n=11) removed | Breakfast, Lunch, Dinner |
| Which one snack would you like to keep as solid? | Morning snack, Afternoon snack, Evening snack | - No answer (n=24) removed | Morning snack, Afternoon snack, Evening snack |
| Would you try EEN? | Yes, No, I don’t know | - “I don’t know” (n=42) merged with “No” (n=35) -> “No”  - No answer (n=3) removed | Yes, No |
| Would you try PEN? | Yes, No, I don’t know | - “I don’t know” (n=18) merged with “No” (n=15) -> “No”  - No answer (n=3) removed | Yes, No |
| Would you prefer strict or flexible meal plan? | Strict plan, flexible plan, I don’t know | - “I don’t know” (n=16) and no answer (n=7) removed | Strict plan, Flexible plan |
| Would you prefer pre-made/ready to drink formula or powder to mix? | Pre-made/ready to drink milkshake, Milkshake powder to mix, I don’t know | - “I don’t know” (n=26) and no answer (n=7) removed | Pre-made/ready to drink milkshake, Milkshake powder to mix |
| Do you think PEN/EEN could help with CD? | Yes, No, I don’t know | - “I don’t know” (n=99) merged with “No” (n=16) -> “No”  - No answer (n=3) removed | Yes, No |
| Do you think diet could help with CD? | Yes, No, I don’t know, I already do this | - “I don’t know” (n=45) merged with “No” (n=7) -> “No”  - “I already do this” (n=21) merged with “Yes” (n=84) -> “Yes”  - No answer (n=3) removed | Yes, No |
| Would you prefer PEN/EEN or food-based dietary therapy with a meal plan to prepare at home? | EN, Food-based dietary therapy meal plan, I don’t know | - “I don’t know” (n=36) and no answer (n=6) removed | PEN/EEN, Food-based dietary therapy meal plan |
| Would you prefer food-based dietary therapy with meal plan to prepare at home or with pre-made meals delivered? | Food-based dietary therapy with meal plan to prepare at home, Food-based dietary therapy with pre-made meals delivered, I don’t know | - “I don’t know” (n=16) and no answer (n=9) removed | Food-based dietary therapy with meal plan to prepare at home, Food-based dietary therapy with pre-made meals delivered |

**Supplementary Table 1:** Data cleaning process for the analyses with chi-square test with post-hoc Fischer’s exact test and Random Forest-Recursive Feature Elimination algorithm.

Abbreviations: CD, Crohn’s disease; EEN, Exclusive Enteral Nutrition; PEN, Partial Enteral Nutrition

| **Predictors** | **Answers** |
| --- | --- |
| Sex | Female; Male |
| Age | 16-24; 25-39; 40-65; >65 |
| Living situation | Living with family members; Living without family members |
| Education level | Secondary school or lower; College/sixth form/trade/technical/vocational training; Higher education |
| Employment status | Employed; Unemployed, Retired |
| Ethnicity | Caucasian; non-Caucasian |
| Exclusion diet use | Yes; No |
| Gluten-free diet | Yes; No |
| Vegetarian diet | Yes; No |
| Low sugar diet | Yes; No |
| Low fibre diet | Yes; No |
| Low spice diet | Yes; No |
| Low food additives diet | Yes; No |
| Low dairy diet | Yes; No |
| Previous liquid meal replacement outside CD | Yes; No |
| Food preparation | Me; Me and another household member; Another household member |
| Any meals/snacks shared | Yes; No |
| EEN exposure | Yes; No |
| PEN exposure | Yes; No |
| Intestinal resection exposure | Yes; No |
| Number of symptoms experienced in the last 7 days | 0; 1; 2; 3; ≥4 |
| Self-reported CD activity | Inactive; Mild to moderate; Severe |
| Time of diagnosis | More than 10 years ago; In the last 10 years |

**Supplementary Table 2:** Demographics data used for the analyses with chi-square test with post-hoc Fischer’s exact test and the Random Forest-Recursive Feature Elimination algorithm.

Abbreviations: CD, Crohn’s disease; EEN, Exclusive Enteral Nutrition; PEN, Partial Enteral Nutrition.

| **Sex**, Female | 53% (85/160) |  | **Living situation** | |
| --- | --- | --- | --- | --- |
| **Age**, years | |  | Living alone | 21% (34/159) |
| <18 | 1% (1/160) |  | Living in a shared accommodation | 4% (7/159) |
| 18-24 | 9% (15/160) |  | Living with a partner | 39% (62/159) |
| 25-39 | 29% (46/160) |  | Living with family members | 35% (56/159) |
| 40-65 | 44% (70/160) |  | **Number of symptoms experienced in the last 7 days** | |
| >65 | 18% (28/160) |  | 0 | 26% (40/156) |
| **Ethnicity** | |  | 1 | 20% (31/156) |
| White British | 88% (141/160) |  | 2 | 15% (23/156) |
| White Other | 6% (9/160) |  | 3 | 19% (29/156) |
| White Irish | 3% (4/160) |  | 4 | 13% (21/156) |
| Pakistani | 2% (3/160) |  | 5 | 6% (9/156) |
| Asian Other | 1% (1/160) |  | >5 | 2% (3/156) |
| White and Asian | 1% (1/160) |  | **Time of diagnosis** | |
| White and Black Caribbean | 1% (1/160) |  | Last year | 3% (5/159) |
| **Education level** | |  | In the last 5 years | 22% (35/159) |
| No schooling completed | 3% (4/148) |  | In the last 10 years | 18% (28/159) |
| Primary school | 1% (1/148) |  | More than 10 years ago | 57% (91/159) |
| Secondary school | 18% (26/148) |  | **Treatment exposures** | |
| College/Sixth form | 20% (29/148) |  | EEN | 21% (33/159) |
| Trade/technical/vocational training | 12% (18/148) |  | PEN | 30% (48/159) |
| Bachelor’s degree | 28% (41/148) |  | Steroids | 87% (138/159) |
| Master’s degree | 14% (21/148) |  | Immunosuppressants | 72% (115/159) |
| Doctorate degree | 5% (8/148) |  | Intestinal resection | 37% (59/159) |
| **Employment/education status** | |  | **Crohn’s disease severity**, self-reported | |
| Full-time employment | 40% (62/154) |  | Inactive | 32% (47/148) |
| Part-time-employment | 15% (23/154) |  | Mild to moderate | 61% (90/148) |
| Full-time education | 9% (14/154) |  | Severe | 7% (11/148) |
| Part-time education | 2% (3/154) |  |  |  |
| Self-employed | 8% (13/154) |  |  |  |
| Stay-at-home parent | 2% (3/154) |  |  |  |
| Retired | 21% (32/154) |  |  |  |
| Unemployed | 6% (10/154) |  |  |  |

**Supplementary Table 3:** Demographics, disease characteristics and treatment exposures of adult patients with Crohn’s disease who returned the survey (n=160).

Abbreviations: EEN: Exclusive Enteral Nutrition; PEN: Partial Enteral Nutrition

| **Questions** | **Answers, % (n)** | | | | | | |
| --- | --- | --- | --- | --- | --- | --- | --- |
|  | Me | My partner | Another household member | Me and another household member | Mostly eat takeaway/fast food | Mostly eat ready microwave meals | Mostly eat restaurant food/dine out |
| Who normally prepares your food? | 64% (100/156) | 17% (27/156) | 9% (14/156) | 7% (11/156) | 1% (2/156) | 1% (1/156) | 1% (1/156) |
|  | Yes | | No | | Not applicable/I live alone | | |
| Do you eat the same meals as the rest of your household? | 63% (96/152) | | 18% (28/152) | | 18% (28/152) | | |
|  | Yes | | No | | | | |
| Have you used liquid meal replacement for a reason other than for CD management? | 18% (28/157) | | 82% (129/157) | | | | |

**Supplementary Table 4:** Eating habits of adult patients with Crohn’s disease.

Abbreviations: CD, Crohn’s disease.

| **Concern category** | **n, %** | **Examples** |
| --- | --- | --- |
| Taste/palatability | 25% (34/136) | “I really don't like milkshakes so would probably struggle to drink them.”; “The taste. I heard the taste was horrible though. I haven't tried yet. If the taste is good, I don't mind” |
| Satiety/hunger | 22% (30/136) | “Probably hunger as I have a very active job + home life and tend to eat little and often.”; “I may start to get hungry and want to snack but feel I would manage.” |
| Taste fatigue | 14% (19/136) | “Possibly becoming fed up with the taste of the milkshakes.”; “I think I might get bored quickly with drinking the shakes every day.” |
| Impact on social life | 14% (19/136) | “Going out with friends and family.”; “The act of sitting down to meal is also as much a social interaction or event. Drinking milkshakes is a relatively perfunctory activity.” |
| Impact on fulfilment from food | 13% (17/136) | “I like making my food and having a break from work at lunch to have lunch.”; “The fulfilment of making and eating food”; “Frustration at not being able to eat normally (favourite foods are one of the joys of life) and the unpleasant taste of drinking specialised milkshake drinks would lower my quality of life.” |
| Effectiveness of treatment | 10% (14/136) | “At age 76, I feel any change would make no difference to my condition.”; “Unsure on how a milkshake will help my Crohn’s.” |
| Impact on family life | 10% (13/136) | “Difficult when I am preparing meals for my family but wouldn't be eating myself.” “My wife would not like cooking for one if I was not eating the same meal as her.” |
| Side effects | 10% (13/136) | “Unsure if this plan would change my toilet motions for better or worse.”; “I also found their flavour (drinks) quite nauseating even when mixed with milkshake mixers e.g., Crusha.” |
| Ingredients in formula | 8% (11/136) | “I try to avoid dairy products.”; “Dietary option of halal ingredients would be a must.” |
| Impact on energy levels | 7% (10/136) | “Low on energy and always feel hungry and tired.”; “I work 12-hour shifts at work. I am also a paramedic student, and work 12 hour shifts on placement also. I think I would find it difficult to maintain my energy levels without my usual amount of food.” |
| No concerns | 7% (10/136) | “None - have been asking for this since diagnosis in 2016! Told it would make no difference so not referred any further. - Very disappointing/frustrating!”; “I am very interested in this proposal and I do think gut health is the key to improvement” |
| Restricted snacking | 6% (8/136) | “Not actively snacking in between.”; “Breaking the habit of snacking. My fatigue and sometimes lack of appetite makes me snack frequently instead of preparing proper meals.” |
| Disruption to routine | 6% (8/136) | “Very difficult as I have a fixed routine which is working for me just now.”; “Adapting my daily routine although when my Crohn's is active, I would do anything!” |
| Impact on mental health | 5% (7/136) | “Could potentially be triggering to people with histories of disordered eating. I would recommend this extreme diet be done alongside adequate psychological support.”; “I've tried liquid supplements during really bad flares when I was diagnosed as a teenager (over 10 years ago), and the effect on my mental health outweighed any possible physical benefits. It was depressing and difficult and made me stop.” |
| Weight maintenance | 5% (7/136) | “Weight loss/gain. I do not consume a large amount of calories usually anyway but I do struggle with my weight.”; “I have always been slim and struggled to put on weight. I would be worried about losing more unnecessary weight that I would struggle to put back on.” |
| Motivation to adhere | 5% (7/136) | “Discipline to keep going for 6 weeks. However it would depend on the severity of the symptoms.”; “I knew if I was told to take them I'm afraid I wouldn't just skip the meals in all honesty.” |

**Supplementary Table 5:** Categories of concerns regarding the use of partial enteral nutrition for Crohn’s disease management, identified through thematic analysis of responses from adult patients with Crohn’s disease. Categories with at least 5% of responders are shown.


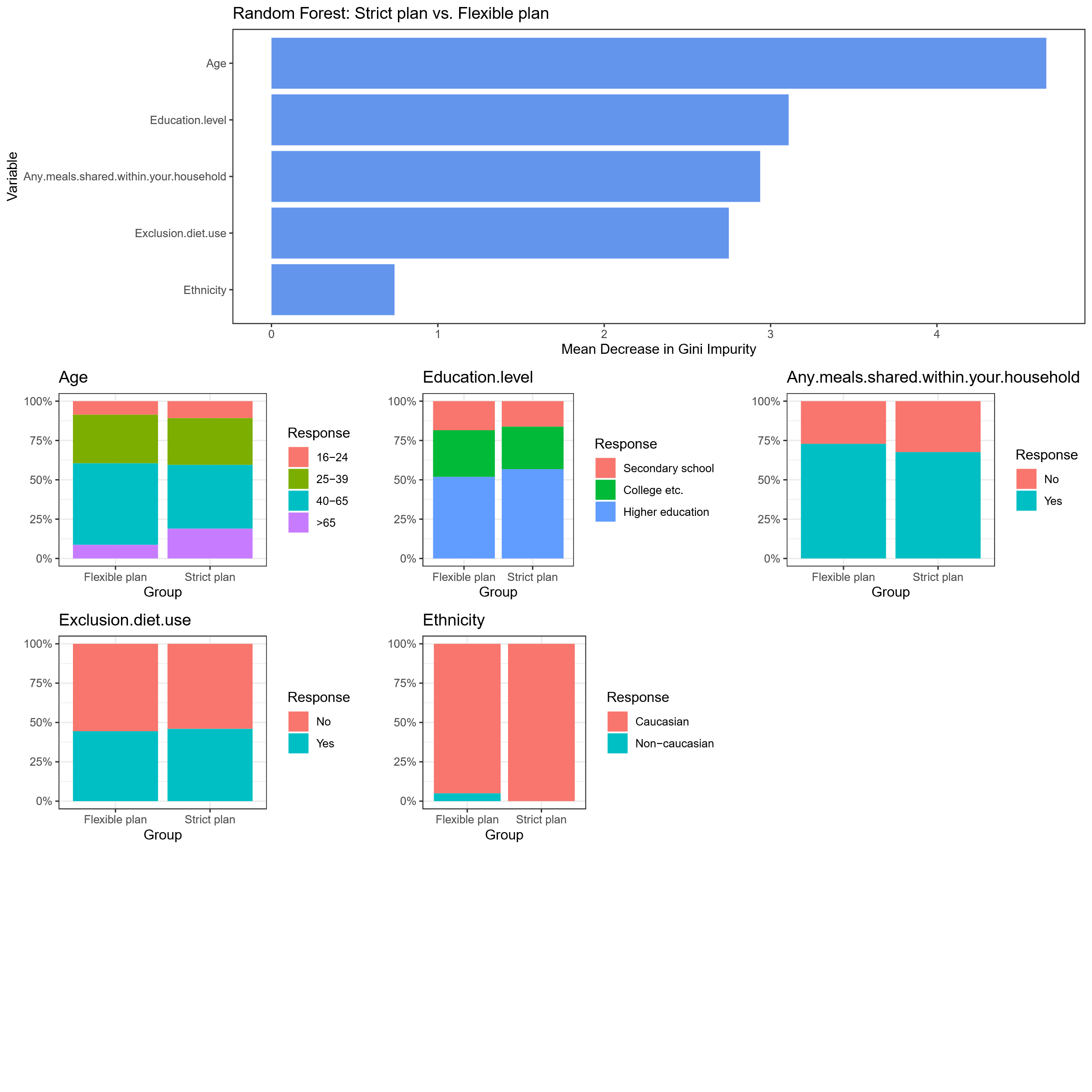


**Supplementary Figure 1:** Results of random forest analysis with mean decrease in Gini impurity score for preference for flexible or strict meal plan with partial enteral nutrition in a subset of patients who would try partial enteral nutrition (n=124) (Area Under the Curve: 0.698; Out-of-bag error: 0.40; P-value: =.976).


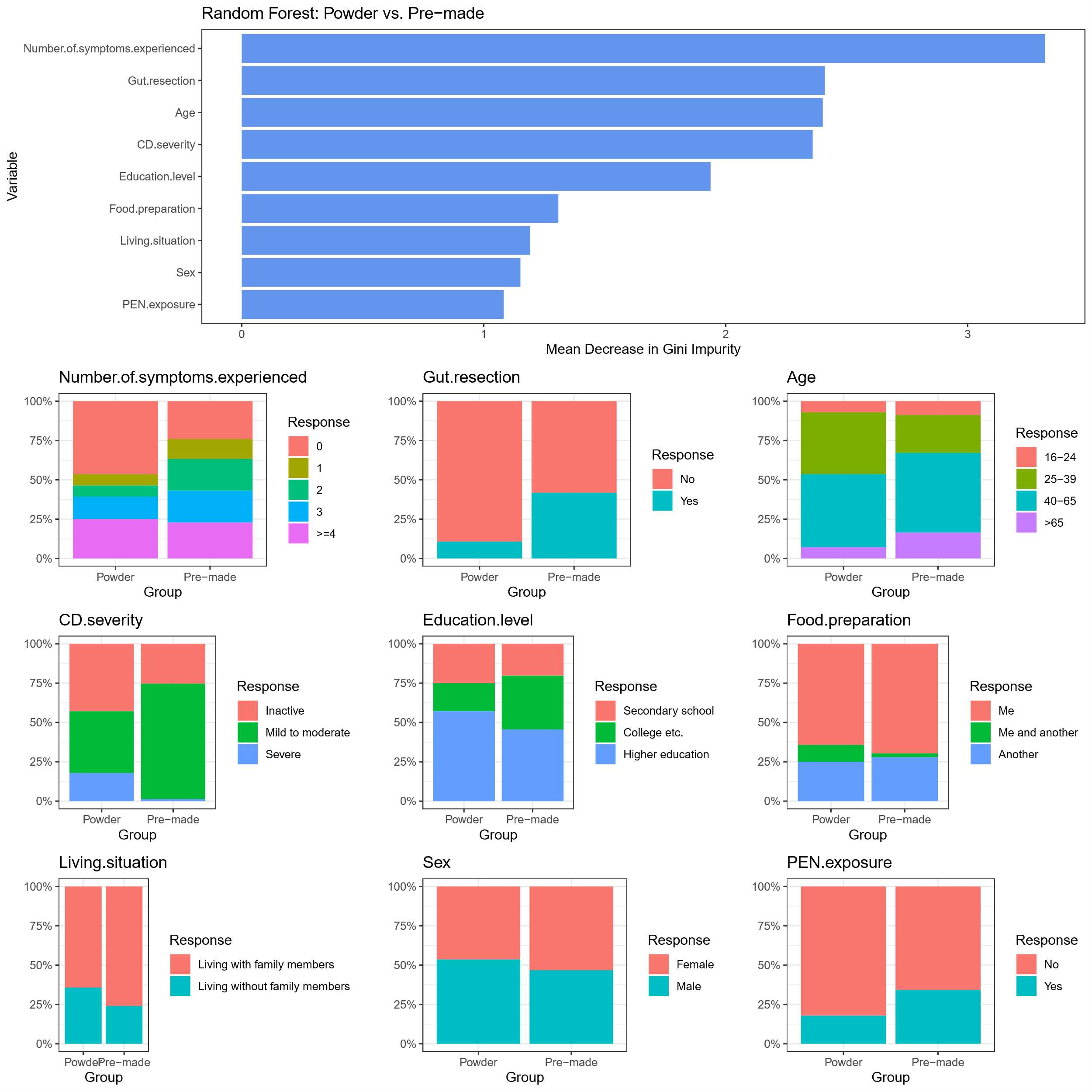


**Supplementary Figure 2:** Results of random forest analysis with mean decrease in Gini impurity score for preference for formula type (powder vs. pre-made) in a subset of patients who would try exclusive enteral nutrition and/or partial enteral nutrition (n=125) (Area Under the Curve: 0.745; Out-of-bag error: 0.70; P-value: =.698).

Abbreviations: CD, Crohn’s Disease; PEN, Partial Enteral Nutrition.


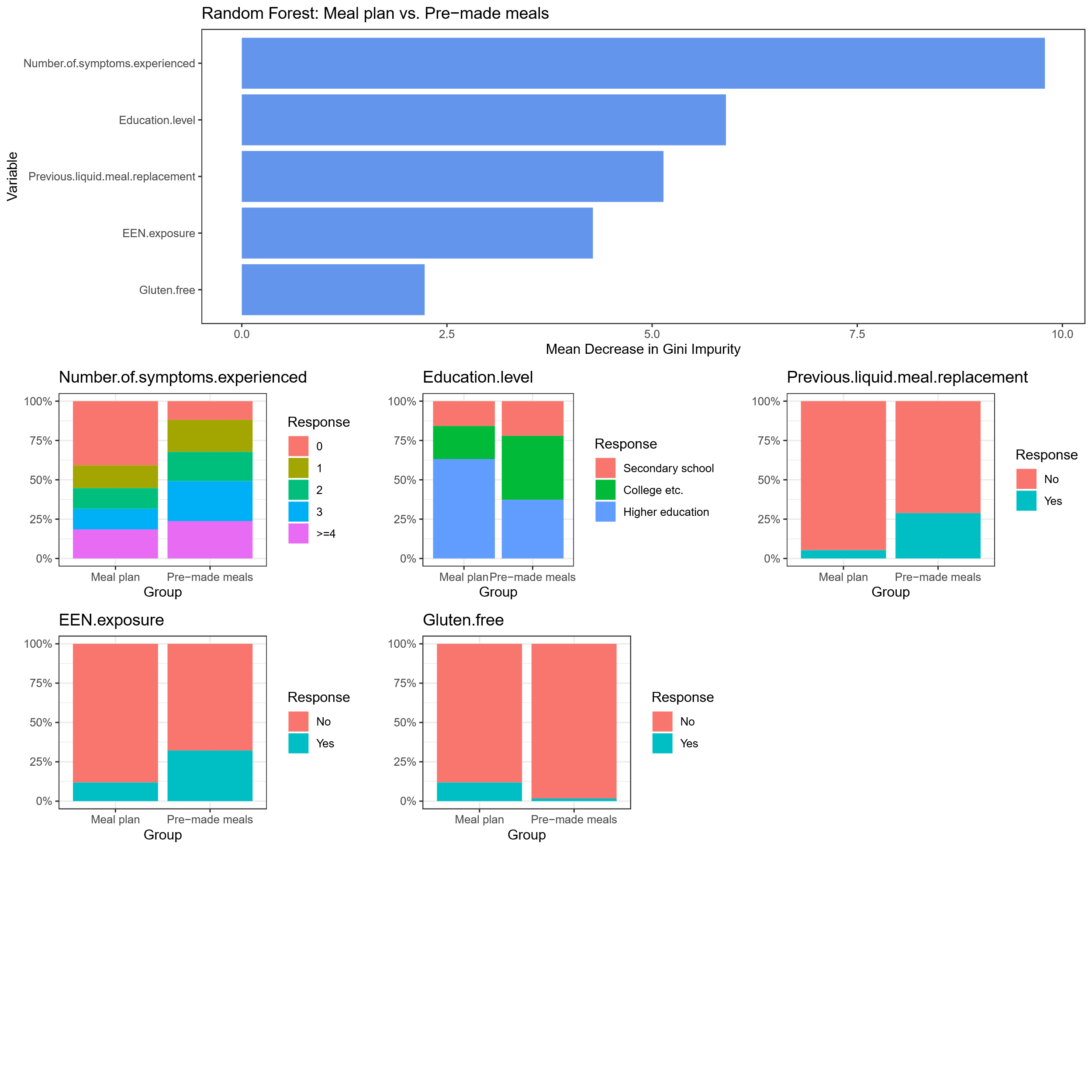


**Supplementary Figure 3:** Results of random forest analysis with mean decrease in Gini impurity score for preference for food-based dietary therapy with a meal plan to prepare at home or with pre-made meals delivered (Area Under the Curve: 0.768; Out-of-bag error: 0.27; P-value: <.001).

Abbreviations: EEN, Exclusive Enteral Nutrition.
